# Skeletal muscle ex vivo mitochondrial respiration parallels decline in vivo oxidative capacity, cardiorespiratory fitness, and muscle strength: The Baltimore Longitudinal Study of Aging

**DOI:** 10.1111/acel.12725

**Published:** 2018-01-21

**Authors:** Marta Gonzalez‐Freire, Paul Scalzo, Jarod D'Agostino, Zenobia A. Moore, Alberto Diaz‐Ruiz, Elisa Fabbri, Ariel Zane, Brian Chen, Kevin G. Becker, Elin Lehrmann, Linda Zukley, Chee W. Chia, Toshiko Tanaka, Paul M. Coen, Michel Bernier, Rafael de Cabo, Luigi Ferrucci

**Affiliations:** ^1^ Longitudinal Studies Section Translational Gerontology Branch National Institute on Aging Baltimore MD USA; ^2^ Clinical Research Unit MedStar Harbor Hospital National Institute on Aging Baltimore MD USA; ^3^ Experimental Gerontology Section Translational Gerontology Branch National Institute on Aging Baltimore MD USA; ^4^ Gene Expression and Genomics Unit National Institute on Aging Baltimore MD USA; ^5^ Diabetes Section Laboratory of Clinical Investigation National Institute on Aging Baltimore MD USA; ^6^ Translational Research Institute for Metabolism and Diabetes Florida Hospital Orlando FL USA

**Keywords:** aging, mitochondria, muscle performance, oxidative capacity, skeletal muscle

## Abstract

Mitochondrial function in human skeletal muscle declines with age. Most evidence for this decline comes from studies that assessed mitochondrial function indirectly, and the impact of such deterioration with respect to physical function has not been clearly delineated. We hypothesized that mitochondrial respiration in permeabilized human muscle fibers declines with age and correlates with phosphocreatine postexercise recovery rate (kPCr), muscle performance, and aerobic fitness. Mitochondrial respiration was assessed by high‐resolution respirometry in saponin‐permeabilized fibers from vastus lateralis muscle biopsies of 38 participants from the Baltimore Longitudinal Study of Aging (BLSA; 21 men, age 24–91 years) who also had available measures of peak oxygen consumption (VO_2max_) from treadmill tests, gait speed in different tasks, ^31^P magnetic resonance spectroscopy, isokinetic knee extension, and grip strength. Results indicated a significant reduction in mitochondrial respiration with age (*p < *.05) that was independent of other potential confounders. Mitochondrial respiratory capacity was also associated with VO_2max_, muscle strength, kPCr, and time to complete a 400‐m walk (*p* < .05). A negative trend toward significance (*p *= .074) was observed between mitochondrial respiration and BMI. Finally, transcriptional profiling revealed a reduced mRNA expression of mitochondrial gene networks with aging (*p* < .05). Overall, our findings reinforce the notion that mitochondrial function declines with age and may contribute to age‐associated loss of muscle performance and cardiorespiratory fitness.

## 1 INTRODUCTION

Mitochondrial function declines with age across multiple tissues, especially those with high energy demand such as skeletal muscle, brain, and heart (González‐Freire et al., 2015; Newgard & Pessin, 2014; Peterson, Johannsen & Ravussin, 2012). This functional decline is associated with substantial reduction in various biochemical properties including respiratory capacity, ATP production, and calcium handling capacity and content, as well as increased apoptosis (González‐Freire et al., 2015). Part of the age‐related decline of mitochondrial function in skeletal muscle is due to a reduction in physical activity and can be rescued by exercise (Broskey et al., 2014; Kent and Fitzgerald, 2016; Larsen et al., 2012; Menshikova et al., 2006). However, physical fitness, which is strongly connected with mitochondrial respiration, declines with aging even in individuals who remain physically active (Distefano et al., 2017; Robinson et al., 2017). Therefore, the mechanisms and the extent by which chronological age “per se” affects the mitochondrial energy production remain unclear.

In previous studies, we assessed muscle mitochondrial function in a relatively large group of participants from the Baltimore Longitudinal Study of Aging (BLSA) using ^31^P magnetic resonance spectroscopy (MRS) and showed that muscle bioenergetics, assessed as postexercise phosphocreatine recovery rate (kPCr), declines with aging and correlates with poorer performance in mobility tasks, especially those performed at fast pace or on long distances (Choi et al., 2016). Consistent with this study and using the same in vivo measurements, Zane et al. (2017) demonstrated that muscle strength significantly mediates the relationship of mitochondrial function with walking performance. However, studies that assessed mitochondrial electron transport chain respiration “ex vivo” by performing assays in permeabilized muscle fibers yielded controversial results concerning the effect of aging as well as correlations with physical capacity tests (Bharadwaj et al., 2015; Coen et al., 2013; Distefano et al., 2017; Tyrrell et al., 2015). This discordance in the literature may be related to inherent methodological differences. ^31^P MRS assesses mitochondrial capacity to generate ATP following muscle contraction in vivo and integrates all aspects of mitochondrial content and function including oxygen consumption, efficiency, and Ca^2+^ handling, which together contribute to produce ATP. ^31^P MRS may be sensitive to factors external to mitochondria such as adequate perfusion and oxygen delivery during and after exercise, changes in intracellular pH and other characteristics of the microenvironment. Therefore, it is unclear whether the decline in oxidative capacity observed in our previous studies is a direct consequence of an intrinsic impairment in the mitochondrial electron transport chain (ETC) with aging. Ex vivo respiration assays in permeabilized muscle fibers may address this problem as they specifically assess ETC function and are conducted in highly controlled conditions including saturating levels of oxygen and substrates.

To explore whether aging is associated with a primary intrinsic dysfunction of mitochondrial ETC respiratory capacity, we performed ex vivo mitochondrial respiration in permeabilized fibers from vastus lateralis muscle biopsies obtained from 38 BLSA participants who also had data available on treadmill peak oxygen consumption (VO_2max_), gait speed in multiple mobility tasks, isokinetic knee extension strength, and postexercise ^31^P MRS. We hypothesized that ex vivo mitochondrial respiration correlates with in vivo measures of oxidative capacity and is also associated with muscle strength and performance in mobility tasks. Finally, we determined whether alterations in mitochondrial gene expression networks could support this changes in the mitochondrial phenotype observed with aging in skeletal muscle.

## 2 RESULTS

### 2.1 Participant characteristics

The main characteristics of the study population, including demographics, physiological, and mitochondrial respiration parameters, are summarized in Table 1 and Table S1.

**Table 1:** Characteristics of the 38 BLSA participants included in the study

Parameters	Values
Age, years	68.55 ± 16.81
Men (%)	21 (55%)
Race (White), *N* (%)	26 (68.3%)
Height, cm	169.28 ± 7.82
Weight, kg	75.49 ± 14.59
BMI, kg/m^2^	26.11 ± 4.06
VO_2max_ (ml/kg/min)	22.85 ± 5.39
DEXA‐Total body fat mass, kg	24.37 ± 10.33
DEXA‐Total body lean mass, kg	48.32 ± 9.03
Quad. Left conc. 30°/sec peak torque (Nm)	127.54 ± 50.97
Maximum grip strength (kg)	33.50 ± 12.70
Usual gait speed (m/s)	1.15 ± 0.20
Rapid gait speed (m/s)	1.81 ± 0.32
400 m fast pace walk (s)	269.58 ± 55.40
*k*_PCr_ (s^−1^)	0.021 ± 0.005

BMI, body mass index; VO_2max_, maximum oxygen consumption; DEXA, total body dual‐energy x‐ray absorptiometry; k_PCr_, phosphocreatine postexercise recovery.

Values are mean ± standard deviation.

### 2.2 Mitochondrial respiration and its association with aging and body composition

**Figure 1:**
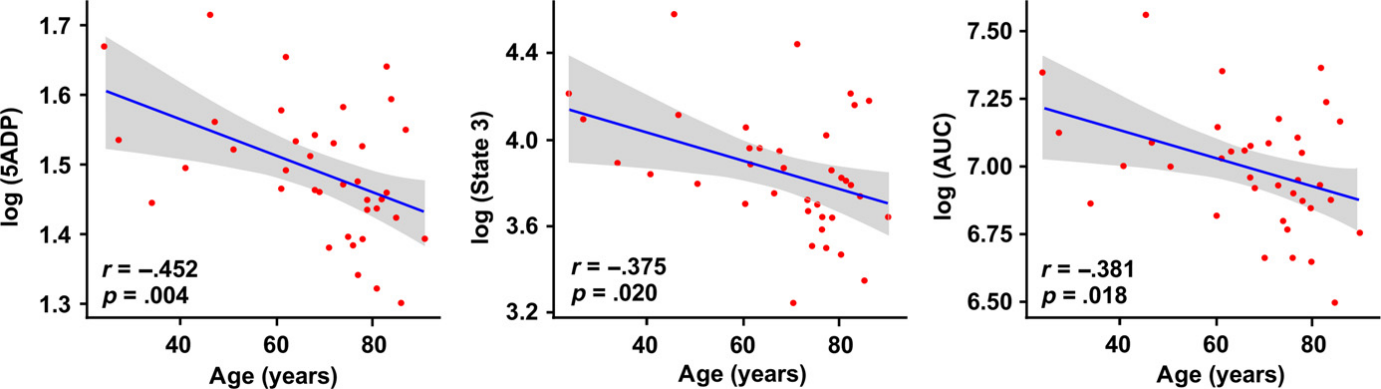
Representative scatterplots showing the association of mitochondrial respiration with age. The association between oxygen consumption at 5ADP (left panel), 2 mm ADP (defined as State 3, center panel), and AUC (right panel) with age is presented after adjustment for sex and BMI. AUC, area under the curve

**Figure 2:**
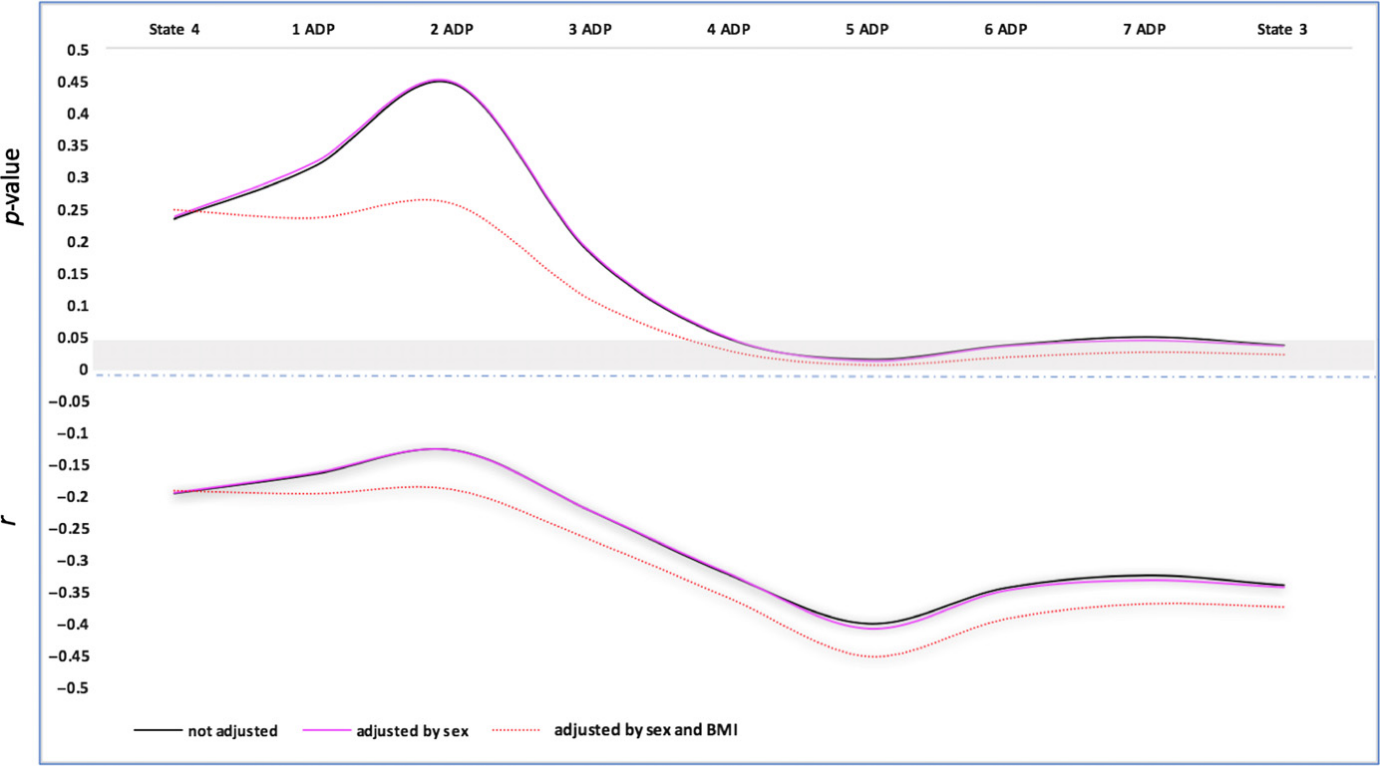
Association of the mitochondrial respiration derived from the different concentrations of ADP in function of age. Line graphs showing the *p*‐values (upper panel) and *r* (bottom panel) of the bivariate association between ADP concentrations and age (model not adjusted, adjusted for sex, and then adjusted for sex and BMI). 1ADP to 8ADP, ADP concentrations of 31.25 μm to 1 mm; State 3: 2 mm; State 4: malate (5 mm), glutamate (10 mm), succinate (10 mm)

Mitochondrial respiration was carried out with permeabilized muscle fiber bundles from 38 participants using the Oxygraph‐2k. The correlations and partial correlations between the different mitochondrial respiration measurements and age are summarized in Table 2. Sex or race did not significantly alter the mitochondrial respiration values, although a slightly higher mitochondrial respiration was observed in men compared to women (data not shown). State 4 respiration, defined as oxygen consumption before the addition of ADP, therefore in the absence of adenylates, and the respiration rate following the addition of the three lowest doses of ADP (31.25–62.5 μm) were not correlated with aging (*p* > .05). However, a statistically significant association between ADP concentrations ranging from 125 μm to 2 mm and aging was found, which was even more evident after adjustment for sex and BMI (Figure 1). The measure of respiration that closely correlated with aging was obtained with 0.25 mm ADP (now referred as 5 ADP or Submaximal State 3) rather than the highest ADP concentration used (2 mm; defined as State 3 respiration; *r* = −.452, *p *= .004 versus *r* = −.375, *p *= .020). The significant difference in respiration between 0.25 and 2 mm ADP occurred independently of age, sex, or BMI (*p *< .05). These findings suggest that with aging, mitochondria become less able to increase oxidative phosphorylation under maximal ADP stimulation, perhaps because of a lower efficiency of the electron transport chain to keep up with the demand (Figure 2). Results were similar when calculating AUC of the entire titration curve from every participant using the trapezoidal formula (see method section). The AUC represents total mitochondrial oxygen consumption over time, for example, the ability to the mitochondria to produce ATP. A statistically significant negative association existed between mitochondrial oxygen consumption and aging that was independent of sex or BMI (*r* = −.381, *p *= .018).

**Table 2:** Correlations between mitochondrial respiration at the different ADP titrations and age as measured by the Oroboros Oxygraph‐2k

	Age		Age^a^		Age^b^	
	*r*	*p*	*r*	p	*r*	*P*
State 4	−.198	.233	−.197	.236	−.193	.246
1 ADP	−.168	.314	−.166	.320	−.198	.233
2 ADP	−.130	.445	−.130	.447	−.191	.256
3 ADP	−.225	.179	−.225	.181	−.270	.106
4 ADP	−.325	.046^c^	−.324	.047^c^	−.361	.026^c^
5 ADP	−.401	.014^c^	−.410	.011^c^	−.452	.004^c^
6 ADP	−.345	.036^c^	−.350	.034^c^	−.393	.016^c^
7 ADP	−.326	.049^c^	−.334	.043^c^	−.370	.024^c^
State 3	−.341	.036^c^	−.345	.034^c^	−.375	.020^c^
AUC	−.326	.046^c^	−.330	.043^c^	−.381	.018^c^

ADP, adenosine diphosphate; AUC, area under the curve.

Pearson and partial correlations are presented.

a Adjusted by sex.

b Adjusted by sex and BMI.

c Statistically significant.

Next, we examined the relationships between mitochondrial respiration and measurements of body composition such as height, weight, BMI, fat mass, and lean body mass. While none of the parameters of mitochondrial respiration were significantly correlated with height, weight, or lean body mass (*p *> .05), a borderline significant inverse association was observed between respiration at Submaximal State 3 and BMI or fat mass (*r* = −.292, *p *= .074; *r* = −.302, *p *= .065, respectively) after adjustment for age and sex (Figure S2).

### 2.3 Mitochondrial respiration and its association with mobility, cardiorespiratory measurements, and muscle function

Significant positive correlations between mitochondrial respiration and VO_2max_, muscle strength, and mitochondrial oxidative capacity were observed, whereas mitochondrial respiration was negatively associated with the time to perform a 400‐m walk (*p *< .05; Table 3; Figure S3). No significant association was found between mitochondrial respiration and gait speed assessed at the 6‐m walking tasks (data not shown).

**Table 3:** Bivariate correlations showing the association between mitochondrial function and cardiorespiratory fitness, muscle function, and in vivo oxidative capacity

	State 4		Submaximal State 3 (5 ADP)		State 3	
	*r*	*p*‐value	*r*	*p*‐value	*r*	*p*‐value
VO_2max_						
Pearson correlation	.402	.012^a^	.610	<.001^a^	.360	.026^a^
Adj. *R* ^2^	.138	.012^a^	.355	<.001^a^	.105	.026^a^
Grip strength						
Pearson correlation	.324	.047^a^	.360	.026^a^	.281	.087
Adj. *R* ^2^	.080	.047^a^	.106	.026^a^	.053	.087
Left leg muscle						
Strength						
Pearson correlation	.298	.069	.411	.010^a^	.356	.028^a^
Adj. *R* ^2^	.064	.069	.146	.010^a^	.103	.028^a^
K_PCr_						
Pearson correlation	.459	.004^a^	.376	.023^a^	.213	.205
Adj. *R* ^2^	.188	.004^a^	.116	.023^a^	.020	.205
Time in 400‐m test						
Pearson correlation	−.344	.037^a^	−.435	.007^a^	−.232	.167
Adj. *R* ^2^	.093	.037^a^	.166	.007^a^	.027	.167

ADP, adenosine diphosphate; VO_2max_, maximum oxygen consumption; k_PCr_, phosphocreatine postexercise recovery rate.

Pearson (*r*) and linear regression (Adj. *R* ^2^) models are presented.

a Statistically significant.

Multiple linear regression models were used to test whether mitochondrial respiration in either State 4, State 3, and Submaximal State 3 was associated with measurements of mobility (gait speed and time to complete the 400‐m walking test), muscle strength (grip strength and knee extension strength), cardiorespiratory fitness (VO_2max_), and ^31^P MRS estimate of mitochondrial oxidative capacity (k_Pcr_) independent of age, sex, and BMI (Table S2). These associations were substantially attenuated after adjustment for sex, age, and BMI, but remained statistically significant when assessing the association between State 4 respiration with VO_2max_ (β = .252, *p *= .028) and mitochondrial k_Pcr_ (β = .390, *p =* .017). The relationship of the Submaximal State 3 (0.25 mm ADP or 5 ADP titration) with VO_2max_ remained also statistically significant (β = .297, *p *= .023).

### 2.4 Age‐associated alterations in the skeletal muscle transcriptome

**Figure 3:**
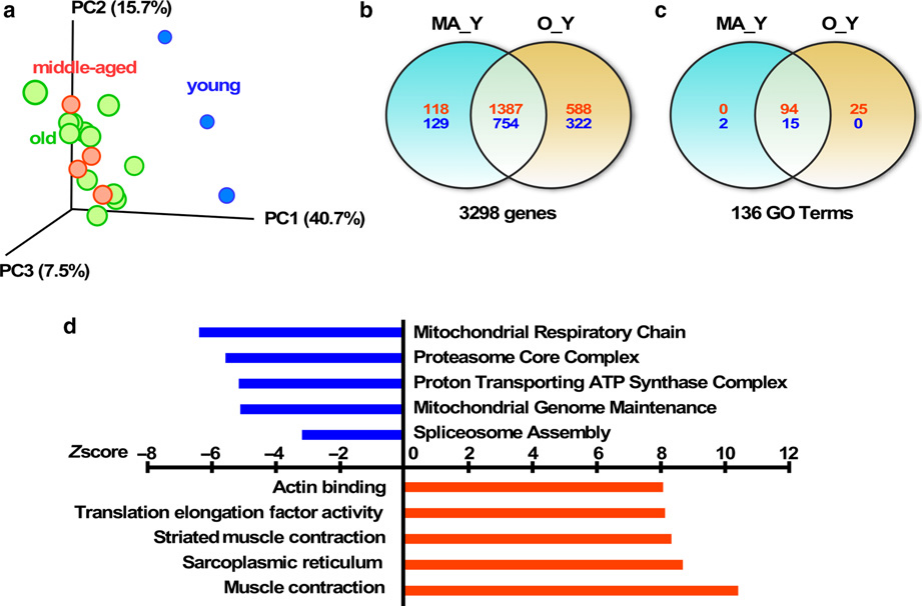
Age‐associated alterations in skeletal muscle transcriptome. (a) Principal component analysis (PCA) showing a clear effect of aging in the skeletal muscle transcriptome among the different age groups. (b) Venn diagram illustrating the number of significantly up‐ (red) and down‐regulated (blue) genes observed in the indicated pairwise comparisons. (c) Venn diagram illustrating the number of significantly up (red)‐ and down‐regulated (blue) GO Terms observed in the indicated pairwise comparisons. (d) Graphical representation of a partial list of significant GO Terms shared between the MA_Y and O_Y pairwise comparisons, with the *Z*‐score values of O_Y depicted. MA, middle‐aged (*N* = 4), O, old (*N* = 12); Y, young (*N* = 3)

Lastly, we tested whether the age‐related decrease in mitochondrial respiration in skeletal muscle was accompanied by a reduced mRNA expression of mitochondrial genes. RNA obtained from the vastus lateralis of a subset of the participants (*N* = 24), aged 25–84 years (mean = 67 ± 15), was analyzed using Illumina microarrays. Five of the samples were removed of the final analysis because of low RNA quality (one young, three middle age, and one old participants). Principal component analysis (PCA) revealed a clear effect of age, especially the young group (PC1, age range: 24–42) when compared to the middle‐aged (age range: 61–67) and old (age range: 73–84) individuals, which clustered together (Figure 3a). To understand the determinants responsible for the differences in skeletal muscle transcriptome with aging, a two‐way Venn diagram was plotted with the middle‐aged versus young (MA_Y) and old versus young (O_Y) pairwise comparisons (Figure 3b). Aging was associated with the significant upregulation of 2093 genes and underrepresentation of 1205 genes. Of note, only three transcripts were uniquely found in the O_MA comparison, indicating the great similarity in the transcriptome of middle‐aged versus old vastus lateralis muscle. This explains why the O_MA pairwise comparison was not incorporated in the Venn diagram. More than 66.3% of significantly upregulated genes (1387/2093) and 62.6% of downregulated genes (754/1205) were shared between the MA_Y and O_Y pairwise comparisons, in support of the PCA analysis that showed clear separation between “young” versus “middle‐aged plus old” cohorts. A list of the top 50 genes is presented in Table S3. Gene ontology analyses of biological processes highlighted 94 out of a total of 136 GO Terms that were shared between MA_Y and O_Y pairwise comparisons (Figure 3c). Of these, “Mitochondrial Respiratory Chain,” “Proteasome Core Complex,” “Proton Transporting ATP Synthase Complex,” and “Mitochondrial Genome Maintenance” were among the top downregulated GO Terms, while “Muscle Contraction” and “Sarcoplasmic Reticulum” were among the top upregulated GO Terms (Figure 3d). The top 10 downregulated genes within “Mitochondrial Respiratory Chain” are depicted in Table S4. A list of all GO Terms in the two pairwise comparisons is presented in Table S5.

## 3 DISCUSSION

In this study, we tested the hypothesis that the intrinsic capacity of the mitochondrial electron transport chain declines with aging. Furthermore, we attempted to link such decline with clinically important measurements of muscle and physical function. Indeed, we found that, independent of sex and BMI, the capacity of the muscle mitochondria to produce energy is significantly lower at older ages. These results are consistent with an age‐associated decline of “in vivo” energetic capacity observed previously in BLSA participants (Choi et al., 2016; Zane et al., 2017). Specifically, we found that at incremental concentrations of ADP, the ability of the mitochondria to produce ATP was diminished in older adults (Figure 2), suggesting that with age mitochondria might lose ability to step up energy production when maximal performance is required, but show relatively normal function when the energetic demand is lower. Such a decline of energy efficiency at a high portion of the energy demand spectrum was previously hypothesized and is consistent with the results of this study, although the molecular mechanisms that explain this observation remain unknown (Porter et al., 2015). Further, we did not find any changes with age in the number of mitochondria (Figure S4); therefore, we believe that the reduction in mitochondrial respiration with age could be due to reduced mitochondrial oxidative phosphorylation efficiency rather than a reduction in mitochondrial number. Although controversial, these mechanisms have been proposed to explain the inability of old mitochondria to step up energy production while showing relatively normal function when the energetic demand is lower. These findings have been already observed in small series of muscle biopsies and may explain the inconsistencies across studies (Coen et al., 2013; Distefano et al., 2017; Tyrrell et al., 2015). We note that the observation of lower energetic capacity at the highest levels of demand is more consistent with intrinsic defect in oxidative phosphorylation when challenged above a certain threshold. For example, studies in mice have found that skeletal muscle mitochondria are organized in a highly reticular structure around the myofibrils (Glancy et al., 2015; Patel, Glancy & Balaban, 2016; Porter et al., 2015). Glancy et al. (2014) have hypothesized that this structure is essential to ensure that mitochondrial function is preserved at the center of the muscle fiber in condition of high energetic demand. A loss of integrity and connectivity of the mitochondrial network would lead to the observed age‐related decline of energetic efficiency and adaptability to challenging conditions.

Recently, we have shown that (i) mitochondrial function correlates with insulin resistance and its severity in nondiabetic older persons, suggesting that decline in mitochondrial function occur in the early stage of development of diabetes type 2 (Fabbri et al., 2017) and (ii) that part of the decline in mitochondrial function is due to reduced perfusion as indicated by lower values of the ankle brachial index (ABI), although within the normal limits (AlGhatrif et al., 2017). Taken together, these two studies suggest that decline in mitochondrial function occur early in the pathways to metabolic dysregulation and disability. They also suggest that the true decline of mitochondria function “in vivo” may be more pronounced from what is measured “in vitro”. Our data might indicate that the decline of intrinsic electron transport chain respiratory capacity with age has functional consequences. In spite of the limited sample size, higher mitochondrial respiration in State 4, submaximal State 3, and State 3 were positively associated with cardiorespiratory fitness, explaining almost 40%–50% of the variance in VO_2max_ when not adjusted for other covariates. After adjustment by sex, age, and BMI, the relationship remained significant, except for respiration in State 3. These findings are consistent with the hypothesis that the loss of mitochondrial function contributes to the decline in VO_2max_ with aging_._ Similar results were published recently by Distefano et al. (2017), but surprisingly these authors could not demonstrate any significant decline of mitochondrial respiration with chronological aging, possibly because of the different characteristics of the population studied by these authors. Both mitochondrial respiration and VO_2max_ are highly affected by the amount of physical activity and exercise of the individual, and therefore, the aging effect could be hidden by the heterogeneity of fitness across age in the studied sample. Thus, we believe that some of the age‐related changes in mitochondrial function might be likely the result of changes in behavior, such as reduced physical activity, and might be muscle type dependent, as it is known that aging affect or modify the movement patterns, and therefore, the different muscles may be affected differently. Previous studies have shown that the tibialis anterior is one of the muscles that is more affected by changes in the gait pattern during aging (Kent and Fitzgerald, 2016).

Mitochondrial respiration was positively associated with muscle strength in both the upper and the lower extremities. In particular, the 5 ADP titration or Submaximal State 3 was the metric most highly correlated (grip strength: *r* = .360, *p *= .026; left knee extension isokinetic strength: *r* = .411, *p *= .010). However, this association was no longer statistically significant when the analysis was adjusted for age and BMI (β = −.025, *p *= .848 and β = .184, *p *= .192, respectively). P^31^ MRI spectroscopy studies have reported a significant correlation between mitochondrial function and muscle strength, suggesting that muscle function mediates the association between mitochondrial function and mobility (Choi et al., 2016; Santanasto et al., 2016; Zane et al., 2017).

We did not find any association between mitochondrial respiration and gait speed, with or without adjustment for covariates. These findings contrast with those reported by Tyrrell et al. (2015) who showed that mitochondria respiration parameters from isolated skeletal muscle mitochondria were associated with gait speed. Also, Choi et al. (2016) found that kPCr, a measurement in vivo of mitochondria function, is a predictor of gait speed in BLSA participants. Again, our findings could be due to the heterogeneity of the effect in the analyzed samples. For example, it has been shown that the mitochondrial function is associated with walking performance in higher functioning active older adults, but not lower functioning sedentary adults (Santanasto et al., 2016). Interestingly, we found a significant negative correlation of respiration in State 4, Submaximal State 3, and State 3 with the 400‐m walk test, a proxy of mobility and fatigability. Similarly to the association between mitochondrial function and muscle strength, the Submaximal State 3 was the parameter most highly correlated with the time needed to complete the 400‐m lap (*r* = −.435, *p *= .007). After adjustment by sex, age, and BMI, these associations were no longer statistically significant, possibly because of the limited sample size of this study.

Similarly to previous studies, we found a nonsignificant negative trend of mitochondrial respiration with BMI, suggesting that mitochondrial respiration might be negatively affected by adiposity or, alternatively, may contribute to increased adiposity (Bharadwaj et al., 2015; Coen et al. 2015). This trend supports the findings from Moore et al. (2014) that muscle quality declines progressively with age and is negatively affected by obesity. Other studies performed in a much larger population should further test the hypothesis that obesity is associated with muscle mitochondrial dysfunction.

Further supporting the idea of a decline in mitochondrial function with aging, we found an underrepresentation of mitochondrial gene expression in skeletal muscle of old BLSA participants. Specifically, pathways enriched in genes involved in mitochondrial respiratory chain and mitochondrial genome maintenance were among the most downregulated during aging. Previous studies have shown reduced expression of genes involved in energy metabolism and essential metabolic pathways as a result of aging, particularly in skeletal muscle (Reznick et al., 2007; Sandri et al., 2004; Su et al., 2015; Zabielski et al. 2016). Notably, the age‐related underrepresentation of the spliceosome pathway in the MA_Y and O_Y pairwise comparisons is of significance as this pathway has been recently found to be negatively impacted in the myonuclei proteome of old mice (Cutler et al., 2017). Interestingly, the upregulation of pathways related with muscle structure and remodeling was also found in our study. Sarcopenia is a process whereby age‐related decline in skeletal muscle mass and strength occurs, with the muscle undergoing morphological changes and physiological alterations mainly due to a reduction in physical activity. This, in turn, results in a remodeling of the motor unit and decline in the number of motor neurons that innervate the muscle fibers, particularly the type II fibers (González‐Freire, de Cabo, Studenski & Ferrucci, 2014). These, together with many other factors such as mitochondrial dysfunction, oxidative stress, inflammation, and lipid infiltration, play a key role in the musculoskeletal impairment that occurs with aging.

**Figure 4:**
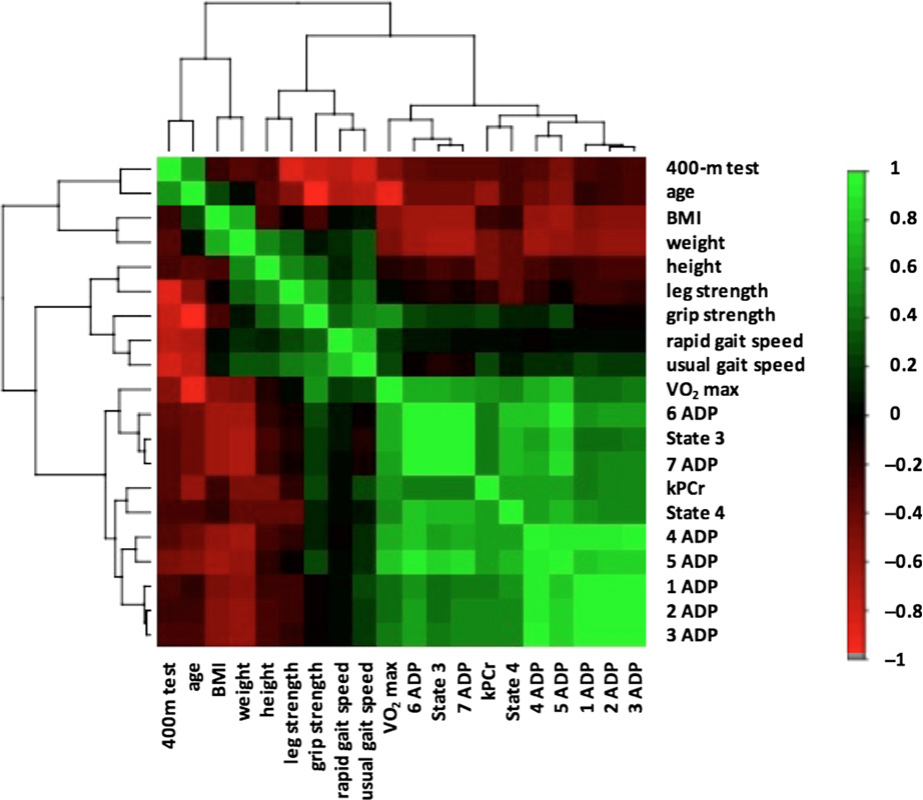
Hierarchical clustering correlation heatmap showing the association between all the covariates included in the analysis. Mitochondrial measurements are clustered with measurements related to oxidative capacity. Positive correlations are displayed in green and negative correlations in red color. Color intensity is proportional to the correlation coefficients

A noteworthy feature of this study is that measurements focused on several aspects of mitochondrial function were found to be significantly correlated. In Figure 4, it is quite clear that all the measurements related to oxidative capacity are well‐clustered together. Indeed, there was a positive association between two distinct mitochondrial functions, namely high‐resolution respirometry and ^31^P magnetic spectroscopy, especially when looking at respiration in State 4 (e.g., respiration in the absence of adenylates [after the addition of malate, glutamate, and succinate]) and in Submaximal State 3. This relationship was maintained after adjustment by sex, age, and BMI only in State 4 respiration (*p* = .017). At this time, we cannot explain the absence of correlation with State 3 respiration. As previously observed for the correlation with age and functional variables, correlation of kPCr with the different ADP titrations becomes smaller with higher concentrations of ADP. These results provide robustness to our findings and might confirm the hypothesis that intrinsic efficiency and capacity of the muscle mitochondria to produce energy decreases with aging and affect muscle performance and cardiorespiratory fitness.

Although our data should be replicated and confirmed in a larger population, we believe that our study strongly suggests that in the search for interventions to slow down muscle aging and to delay or prevent its negative physiological consequences, we should focus our efforts on those interventions that help to maintain or even enhance mitochondrial function in order to prevent its functional decline as we age.

The authors acknowledge the limitations of the study to assess mitochondrial function. However, the substrates and protocols used here (i.e., evaluating State 4 without using oligomycin) are commonly used in the published literature (Oroboros Oxygraph Bioblast: http://wiki.oroboros.at).

## 4 EXPERIMENTAL PROCEDURES

### 4.1 Participants

The Baltimore Longitudinal Study on Aging (BLSA) is a study of human aging established in 1958 and currently supported by the National Institute on Aging (NIA). The design and description of the study have been previously reported (Shock et al., 1984 1984; Stone & Norris, 1966). Adult volunteers age 20 years and older are recruited from the community and healthy at study entry. BLSA participants come in for study visits at age‐specific intervals, undergo medical and various physiological testings at each visits, and are followed for life. The percutaneous muscle biopsy was recently introduced in BLSA. Between August 2013 and June 2016, muscle biopsy specimens were obtained in 41 participants along with measures of mitochondrial respiration by high‐resolution respirometry immediately after the biopsy. Of these, 38 (21 men) aged 24–91 years (mean age 68.6 ± 16.8 years) had complete data from a physical examination, whole‐body aerobic capacity (VO_2max_), body composition assessment, gait speed in different tasks, and muscle function.

### 4.2 Skeletal muscle biopsy procedure

Skeletal muscle biopsies were performed in the morning. The depth of the subcutaneous fat (uncompressed and compressed) was determined from MRI images performed on the previous day. A region above the vastus lateralis muscle was marked at the middle point of an ideal line drawn between the great trochanter and the mid‐patella upper margin. The skin was prepped with povidone–iodine (Betadine^®^) and ethyl alcohol, and the outside areas covered with sterile drapes. The biopsy site was injected intradermally and subcutaneously with about 2 cc of 2% Lidocaine. Then, a 22 gauge × 1 1/2 inch needle was introduced progressively just above the fascia (either sensed by the operator or at the depth indicated on the MRI image). This method ensured that the subcutaneous tissue and muscle fascia were anesthetized without distorting the muscle sample to be biopsied. A 6‐mm Bergstrom biopsy needle was inserted through the skin in the muscle. Multiple muscle specimens were obtained until at least 250 mg of tissue was collected. A small portion of muscle tissue (~15 mg) was immediately placed in ice‐cold respirometry buffer (BIOPS) for mitochondrial respiration analysis (see next section). The rest of the biopsy specimen was snap frozen in liquid nitrogen and subsequently stored at −80°C until used for further analyses.

### 4.3 High‐resolution respirometry

The muscle bundles were carefully dissected in ice‐cold BIOPS buffer to eliminate intermuscular adipose and connective tissue using a dissecting microscope and sharp Dumont forceps. The fiber bundles were then permeabilized in freshly prepared saponin solution (5 μg/ml in BIOPS buffer) for 30 min on wet ice with agitation after which they were transferred to a culture dish plate containing 2 ml buffer Z + EGTA on ice (3 × 10 min). Composition of buffer Z and BIOPS buffer was the same as previously described by Distefano et al. (2017). The wet weight of the fiber bundles was measured for data normalization. The fiber bundles were placed into the chambers of the Oxygraph‐2k (O2k, Oroboros Instruments, Innsbruck, Austria) after air calibration. A summary of the protocol is shown in Figure S1. The light in the chambers was turned off, and blebbistatin (Bleb), a myosin II ATPase inhibitor, capable of blocking spontaneous contraction of fiber bundles and mitigating the effects of contraction in respiratory kinetics (Perry et al., 2011), was added to each chamber. After signal stabilization (~10–15 min), malate (5 mm), glutamate (10 mm), and succinate (10 mm) were added followed by the addition of 8 ADP titrations in increasing amounts (starting at 31.25 μm) until a final concentration of 2 mm, a concentration that we previously tested is saturating and elicits maximal State 3 respiration. Finally, cytochrome C (10 μm) was added at the end of the ADP titration curve to check the integrity of the outer mitochondrial membrane. The fact that respiration did not increase by more than 15% indicated the lack of damage of the outer membrane. The oxygen flux levels were collected using DatLab 4 software (OROBOROS Instruments). Different measurements were performed to determine the function of the mitochondria: State 4 or Leak State (respiration after addition of malate, glutamate, and succinate), State 3 (the theoretical OXPHOS respiration), and respiration at each of the ADP concentrations. Using the trapezoidal formula, the area under the curve (AUC) was calculated to examine the efficiency of the ADP response from the fiber bundles of every participant. To obtain flux in SI units, the “bioenergetic units” [ng atom O∙min^−1^∙mg^−1^ = ng atom O∙min^−1^∙mg^−1^ = μmol O∙min^−1^∙g^−1^] was converted by the multiplication factor [nmol O_2_∙s^−1^∙g^−1^ = pmol O_2_∙s^−1^∙mg^−1^]: 1000/(2∙60) = 8.33 (Gnaiger, 2009).

### 4.4 Mobility measurements

Details of the 400‐m walk test were described elsewhere, and none of the participants had medical contraindications for this test (Newman et al., 2006; Simonsick, Montgomery, Newman, Bauer & Harris, 2001). Briefly, participants were instructed to walk for ten 40‐m laps as quickly as possible receiving feedback and encouragement after completion of each lap. All participants completed the test. Lap time was recorded after the completion of each lap using a stopwatch. Usual and rapid gait speed were measured on a 6‐m course in an uncarpeted corridor. Participants were asked to walk at their usual, comfortable pace and then at the fastest possible pace. Times to complete the 6‐m course were used in the analysis.

### 4.5 Muscle strength

Muscle strength was assessed both as grip strength and as knee extension isokinetic torque. Briefly, grip strength was measured by a Smedley hand dynamometer, calibrated to known weights, and adjusted for hand comfort and fit. Subjects were told to place their arms in a relaxed, stationary position. Three maximal grips were taken, and the highest was recorded for each hand. Maximum quadriceps muscle strength was defined as the highest of three consecutive values of torque (Nm) measured by left leg concentric knee extensor contraction at an angular velocity of 30° per second using an isokinetic dynamometer (Biodex Multi‐Joint System‐PRO with Advantage Software V.4X, Biodex Medical Systems, Inc., Shirley, NY, USA; Hartmann, Knols, Murer & de Bruin, 2009). This torque value is equal to the force generated during the knee extension, multiplied by the length from the knee center to the point where the dynamometer is applied on the tibia.

### 4.6 Body composition

Body composition was determined using dual‐energy x‐ray absorptiometry (DEXA) and computerized tomography (CT). Total body DEXA was performed using a Prodigy Scanner (GE, Madison, WI, USA) and analyzed with version 10.51.006 software. Total nonbone, nonadipose tissue, and adipose tissue at mid‐femur were estimated from a cross‐sectional 10 mm CT image using a Somatom Sensation 10 computed tomography scanner (Siemens, Malvern, PA, USA) and quantified using Geanie software version 2.1 (BonAlyse Oy, Jyvaskyla, Finland). Finally, body mass index (BMI) was calculated as weight (kilograms) divided by the square of height (meters), both measured according to standard protocols.

### 4.7 Cardiorespiratory fitness

Whole‐body aerobic capacity was determined by continuous measurement of oxygen consumption during a modified version of the treadmill Balke protocol (Fleg et al., 2005). Consumed O_2_ and expired CO_2_ concentrations were measured with a gas exchange analyzer system (Ultima C2, MedGraphics, St. Paul, MN, USA). Oxygen consumption expressed in mL/kg/min was calculated every 30 s, and the highest value was considered as the peak VO_2_. The initial incline was 0% and was progressively increased by 3% at each stage until voluntary exhaustion. The first incline change was implemented at 45 s, and all subsequent increases occurred every 3 min until the participants reached a steady state. Peak VO_2_ was the measure of aerobic capacity used in this study.

### 4.8 Skeletal muscle oxidative capacity determined by ^31^P‐MRS

In vivo ^31^P‐MRS measurements of phosphorus‐containing metabolites were obtained from the quadriceps muscles with a 3T Philips Achieva MR scanner (Philips, Best, the Netherlands) using a method previously described (Choi et al., 2016; Zane et al., 2017). Briefly, the participants performed a rapid and intense ballistic knee extension exercise and a series of ^31^P‐MRS spectra were acquired before, during, and after the exercise using a 10‐cm ^31^P‐tuned, flat surface coil (PulseTeq, Surrey, UK) secured over the vastus lateralis muscle of the left thigh. The duration of exercise was controlled to achieve depletion in phosphocreatine (PCr) signal amplitude to a value within 33% to 67% of the resting amplitude. Spectra were processed using jMRUI (version 5.0) and quantified using a nonlinear least square algorithm (AMARES; Naressi, Couturier, Castang, de Beer & Graveron‐Demilly, 2001). Postexercise PCr recovery rate was calculated by fitting the time‐dependent changes in PCr peak area to the monoexponential recovery function: PCr(*t*) = PCr_0_ + ΔPCr (1 − *e* ^−t/τ^) where PCr_0_ is the PCr signal immediately following the exercise during the recovery phase of the protocol, ΔPCr = (PCr at baseline − PCr_0_) is the decrease in PCr signal from the pre‐exercise baseline value to the PCr_0_ value, and τ is the PCr exponential recovery time constant. The inverse of τ_PCr,_ *k*_PCr_ is the PCr recovery rate constant determined as 1/τ.

### 4.9 Immunoblotting

Muscle homogenates were processed for Western blot as previously reported (Distefano et al., 2017). Briefly, proteins were separated by gel electrophoresis using a 4%–20% gel (Bio‐Rad, Mini‐PROTEAN^®^ TGX™ Precast Gel) and transferred onto polyvinylidene difluoride membranes (Bio‐Rad Laboratories, Hercules, CA, USA). Membranes were stained in 5% Ponceau S (Sigma Aldrich), then destained in PBS‐Tween‐20, blocked in 5% nonfat milk, and incubated with the OXPHOS antibody cocktail Abcam (ab110412; 1:5000). After that, the membranes were incubated with the appropriate species‐specific HRP‐conjugated secondary antibodies (Cell Signaling, Danvers, MA, USA). Bands were visualized using chemiluminescent (ECL) Plus Western blotting detection system (GE Healthcare, Piscataway, NJ, USA). Protein loading was controlled by normalizing to the Ponceau S.

### 4.10 Microarray analysis

Total RNA was extracted with TRIzol^®^ (Invitrogen, Carlsbad, CA, USA) from the vastus lateralis of 24 of the 38 participants (age 25–84 years; mean 67 ± 15). Total RNA was quality‐controlled using the Agilent Bioanalyzer RNA 6000 Chip (Agilent, Santa Clara, CA, USA) and labeled according to the manufacturer's instructions using the Illumina^®^ TotalPrep™ RNA amplification kit. A total of 750 ng biotinylated RNA was hybridized to human HT‐12v4 BeadChips (Illumina, San Diego, CA, USA). Following posthybridization rinses, arrays were incubated with streptavidin‐conjugated Cy3 and scanned using an Illumina iScan scanner at 0.54 micron resolution. Hybridization intensity data were extracted from the scanned images using Illumina GenomeStudio software, V2011.1. Raw data were subjected to Z‐normalization, as described elsewhere (Cheadle, Cho‐Chung et al., 2003; Cheadle, Vawter et al., 2003). Principal component analysis (PCA) was performed on the normalized *Z*‐scores of all of the detectable probes in the samples using DIANE 6.0 software (Cheadle, Vawter, Freed & Becker, 2003). Significant genes were selected by the *z*‐test <0.05, false discovery rate <0.30, as well as *z* ratio >1.5 in both directions and ANOVA *p‐*value <.05. Parametric analysis of gene set enrichment (PAGE; Broad Institute, M.I.T., Cambridge, MA) was analyzed as previously described (Kim & Volsky, 2005). All raw data are available in the Gene Expression Omnibus database (Accession No. GSE98613).

### 4.11 Statistical analysis

Distributions of population characteristics were examined through histograms and boxplots. Characteristics of the population are reported as mean ± standard deviation (SD) or percentage. Bivariate correlations (Pearson correlations, partial correlations and linear regressions) were performed to test the relationships between the different mitochondrial respiration measurements with age and the other covariates of the analysis. Multiple linear regressions were used to evaluate the relationship between the three most significant measurements of mitochondrial function (Model 1: State 4; Model 2: 5ADP; and Model 3: State 3) and the different outcomes: fitness (VO_2_max, Grip Strength, Leg Strength), kPCr, and mobility measurements (time in 400 m) independent of age, sex, and body composition. All linear regression coefficients were standardized so as to be comparable (Table S2). Hierarchical clustering correlation heatmap was used to show the association between all the covariates included in the analysis. Hierarchical clustering is used to classify the different outcomes based on their similarity into groups (clusters). This similarity is computed according to a distance between variables (correlation‐based distances). The heatmap represents the Pearson correlation matrix across the different covariates included in the study. All analyses were performed using R version 3.3.1 (R Foundation for Statistical Computing, Vienna, Austria).

## CONFLICT OF INTEREST

The authors declare no competing financial interests.

## AUTHOR CONTRIBUTIONS

MGF, LZ, CWC, PMC, MB, RDC, and LF were involved in the study conception, design, and experimental protocol; MGF, PS, JDA, ADR, ZAM, EF, AZ, TT, BC, KGB, EL, and LF collected the data and helped with the data analysis. MGF and LF wrote the manuscript. All the authors commented, reviewed, and approved the final version of the manuscript.

## ACCESSION CODES

Microarray data have been deposited in the Gene Expression Omnibus database under accession code GSE98613.

## References

[acel12725-bib-0001] AlGhatrif, M. , Zane, A. , Oberdier, M. , Canepa, M. , Studenski, S. , Simonsick, E. , … Ferrucci, L. (2017). Lower mitochondrial energy production of the thigh muscles in patients with low‐normal ankle‐brachial index. Journal of the American Heart Association, 6(9), e006604 https://doi.org/10.1161/JAHA.117.006604 28855165 10.1161/JAHA.117.006604 PMC5634302

[acel12725-bib-0002] Bharadwaj, M. S. , Tyrrell, D. J. , Leng, I. , Demons, J. L. , Lyles, M. F. , Carr, J. J. , … Molina, A. J. A. (2015). Relationships between mitochondrial content and bioenergetics with obesity, body composition and fat distribution in healthy older adults. BMC Obesity, 2, 40 https://doi.org/10.1186/s40608-015-0070-4 26448868 10.1186/s40608-015-0070-4 PMC4594906

[acel12725-bib-0003] Broskey, N. T. , Greggio, C. , Boss, A. , Boutant, M. , Dwyer, A. , Schlueter, L. , … Amati, F. (2014). Skeletal muscle mitochondria in the elderly: Effects of physical fitness and exercise training. Journal of Clinical Endocrinology and Metabolism, 99, 1852–1861. https://doi.org/10.1210/jc.2013-3983 24438376 10.1210/jc.2013-3983

[acel12725-bib-0004] Cheadle, C. , Cho‐Chung, Y. S. , Becker, K. G. , & Vawter, M. P. (2003). Application of z‐score transformation to Affymetrix data. Applied Bioinformatics, 2, 209–217. 15130792

[acel12725-bib-0005] Cheadle, C. , Vawter, M. P. , Freed, W. J. , & Becker, K. G. (2003). Analysis of microarray data using Z score transformation. The Journal of Molecular Diagnostics: JMD, 5, 73–81. https://doi.org/10.1016/S1525-1578(10)60455-2 12707371 10.1016/S1525-1578(10)60455-2 PMC1907322

[acel12725-bib-0006] Choi, S. , Reiter, D. A. , Shardell, M. , Simonsick, E. M. , Studenski, S. , Spencer, R. G. , … Ferrucci, L. (2016). 31P magnetic resonance spectroscopy assessment of muscle bioenergetics as a predictor of gait speed in the Baltimore longitudinal study of aging. Journals of Gerontology. Series A, Biological Sciences and Medical Sciences, 71, 1638–1645. https://doi.org/10.1093/gerona/glw059 10.1093/gerona/glw059 PMC5106855 27075894

[acel12725-bib-0007] Coen, P. M. , Jubrias, S. A. , Distefano, G. , Amati, F. , Mackey, D. C. , Glynn, N. W. , … Goodpaster, B. H. (2013). Skeletal muscle mitochondrial energetics are associated with maximal aerobic capacity and walking speed in older adults. Journals of Gerontology. Series A, Biological Sciences and Medical Sciences, 68, 447–455. https://doi.org/10.1093/gerona/gls196 10.1093/gerona/gls196 PMC3593613 23051977

[acel12725-bib-0008] Coen, P. M. , Menshikova, E. V. , Distefano, G. , Zheng, D. , Tanner, C. J. , Standley, R. A. , … Goodpaster, B. H. (2015). Exercise and weight loss improve muscle mitochondrial respiration, lipid partitioning, and insulin sensitivity after gastric bypass surgery. Diabetes, 64, 3737–3750. https://doi.org/10.2337/db15-0809 26293505 10.2337/db15-0809 PMC4613980

[acel12725-bib-0009] Cutler, A. A. , Dammer, E. B. , Doung, D. M. , Seyfried, N. T. , Corbett, A. H. , & Pavlath, G. K. (2017). Biochemical isolation of myonuclei employed to define changes to the myonuclear proteome that occur with aging. Aging Cell, 16(4), 738–749. https://doi.org/10.1111/acel.12604 28544616 10.1111/acel.12604 PMC5506426

[acel12725-bib-0010] Distefano, G. , Standley, R. A. , Dube, J. J. , Carnero, E. A. , Ritov, V. B. , Stefanovic‐Racic, M. , … Coen, P. M. (2017). Chronological age does not influence ex‐vivo mitochondrial respiration and quality control in skeletal muscle. Journals of Gerontology. Series A, Biological Sciences and Medical Sciences, 72, 535–542. 10.1093/gerona/glw102 PMC6075361 27325231

[acel12725-bib-0011] Fabbri, E. , Chia, C. W. , Spencer, R. G. , Fishbein, K. W. , Reiter, D. A. , Cameron, D. , … Ferrucci, L. (2017). Insulin resistance is associated with reduced mitochondrial oxidative capacity measured by 31P‐magnetic resonance spectroscopy in participants without diabetes from the Baltimore longitudinal study of aging. Diabetes, 66(1), 170–176. https://doi.org/10.2337/db16-0754 27737951 10.2337/db16-0754 PMC5204309

[acel12725-bib-0012] Fleg, J. L. , Morrell, C. H. , Bos, A. G. , Brant, L. J. , Talbot, L. A. , … Lakatta, E. G. (2005). Accelerated longitudinal decline of aerobic capacity in healthy older adults. Circulation, 112, 674–682. https://doi.org/10.1161/CIRCULATIONAHA.105.545459 16043637 10.1161/CIRCULATIONAHA.105.545459

[acel12725-bib-0013] Glancy, B. , Hartnell, L. M. , Malide, D. , Yu, Z.‐X. , Combs, C. A. , Connelly, P. S. , … Balaban, R. S. (2015). Mitochondrial reticulum for cellular energy distribution in muscle. Nature, 523, 617–620. https://doi.org/10.1038/nature14614 26223627 10.1038/nature14614 PMC6988728

[acel12725-bib-0014] Glancy, B. , Hsu, L.‐Y. , Dao, L. , Bakalar, M. , French, S. , Chess, D. J. , … Balaban, R. S. (2014). In vivo microscopy reveals extensive embedding of capillaries within the sarcolemma of skeletal muscle fibers. Microcirculation, 21, 131–147. https://doi.org/10.1111/micc.12098 25279425 10.1111/micc.12098 PMC4185432

[acel12725-bib-0015] Gnaiger E. (Ed.) (2009). Mitochondrial pathways and respiratory control (2nd ed., pp. 38–42). Innsbruck, Austria: OROBOROS MiPNet Publications.

[acel12725-bib-0016] González‐Freire, M. , de Cabo, R. , Bernier, M. , Sollott, S. J. , Fabbri, E. , Navas, P. , & Ferrucci, L. (2015). Reconsidering the role of mitochondria in aging. Journals of Gerontology. Series A, Biological Sciences and Medical Sciences, 70, 1334–1342. https://doi.org/10.1093/gerona/glv070 10.1093/gerona/glv070 PMC4612387 25995290

[acel12725-bib-0017] González‐Freire, M. , de Cabo, R. , Studenski, S. A. , & Ferrucci, L. (2014). The neuromuscular junction: Aging at the crossroad between nerves and muscle. Frontiers in Aging Neuroscience, 6, 208 https://doi.org/10.3389/fnagi.2014.00208 25157231 10.3389/fnagi.2014.00208 PMC4127816

[acel12725-bib-0018] Hartmann, A. , Knols, R. , Murer, K. , & de Bruin, E. D. (2009). Reproducibility of an isokinetic strength‐testing protocol of the knee and ankle in older adults. Gerontology, 55, 259–268. https://doi.org/10.1159/000172832 18997454 10.1159/000172832

[acel12725-bib-0019] Kent, J. A. , & Fitzgerald, L. F. (2016). In vivo mitochondrial function in aging skeletal muscle: Capacity, flux, and patterns of use (2016). Journal of Applied Physiology, 121(4), 996–1003. https://doi.org/10.1152/japplphysiol.00583.2016 27539499 10.1152/japplphysiol.00583.2016

[acel12725-bib-0020] Kim, S. Y. , & Volsky, D. J. (2005). PAGE: Parametric analysis of gene set enrichment. BMC Bioinformatics, 6, 144 https://doi.org/10.1186/1471-2105-6-144 15941488 10.1186/1471-2105-6-144 PMC1183189

[acel12725-bib-0021] Larsen, S. , Hey‐Mogensen, M. , Rabol, R. , Stride, N. , Helge, J. W. , & Dela, F. (2012). The influence of age and aerobic fitness: Effects on mitochondrial respiration in skeletal muscle. Acta Psychologica, 205, 423–432. 10.1111/j.1748-1716.2012.02408.x 22212519

[acel12725-bib-0023] Menshikova, E. V. , Ritov, V. B. , Fairfull, L. , Ferrell, R. E. , Kelley, D. E. , & Goodpaster, B. H. (2006). J Effects of exercise on mitochondrial content and function in aging human skeletal muscle. Journals of Gerontology. Series A, Biological Sciences and Medical Sciences, 61(6), 534–540. https://doi.org/10.1093/gerona/61.6.534 10.1093/gerona/61.6.534 PMC1540458 16799133

[acel12725-bib-0024] Moore, A. Z. , Caturegli, G. , Metter, E. J. , Makrogiannis, S. , Resnick, S. M. , Harris, T. B. , & Ferrucci, L. (2014). Difference in muscle quality over the adult life span and biological correlates in the Baltimore Longitudinal Study of Aging. Journal of the American Geriatrics Society, 62, 230–236. https://doi.org/10.1111/jgs.12653 24438020 10.1111/jgs.12653 PMC3945403

[acel12725-bib-0025] Naressi, A. , Couturier, C. , Castang, I. , de Beer, R. , & Graveron‐Demilly, D. (2001). Java‐based graphical user interface for MRUI, a software package for quantitation of in vivo/medical magnetic resonance spectroscopy signals. Computers in Biology and Medicine, 31, 269–286. https://doi.org/10.1016/S0010-4825(01)00006-3 11334636 10.1016/s0010-4825(01)00006-3

[acel12725-bib-0026] Newgard, C. B. , & Pessin, J. E. (2014). Recent progress in metabolic signaling pathways regulating aging and life span. Journals of Gerontology. Series A, Biological Sciences and Medical Sciences, 69(Suppl 1), S21–S27. https://doi.org/10.1093/gerona/glu058 10.1093/gerona/glu058 PMC4022126 24833582

[acel12725-bib-0027] Newman, A. B. , Simonsick, E. M. , Naydeck, B. L. , Boudreau, R. M. , Kritchevsky, S. B. , Nevitt, M. C. , … Harris, T. B. (2006). Association of long‐distance corridor walk performance with mortality, cardiovascular disease, mobility limitation, and disability. JAMA, 295, 2018–2026. https://doi.org/10.1001/jama.295.17.2018 16670410 10.1001/jama.295.17.2018

[acel12725-bib-0028] Oroboros Instruments . Retrieved from http://wiki.oroboros.at/index.php

[acel12725-bib-0029] Patel, K. D. , Glancy, B. , & Balaban, R. S. (2016). The electrochemical transmission in I‐Band segments of the mitochondrial reticulum. Biochimica et Biophysica Acta, 1857, 1284–1289. https://doi.org/10.1016/j.bbabio.2016.02.014 26921810 10.1016/j.bbabio.2016.02.014 PMC4893892

[acel12725-bib-0030] Perry, C. G. R. , Kane, D. A. , Lin, C.‐T. , Kozy, R. , Cathey, B. L. , Lark, D. S. , … Neufer, P. D. (2011). Inhibiting myosin‐ATPase reveals a dynamic range of mitochondrial respiratory control in skeletal muscle. Biochemical Journal, 437, 215–222. https://doi.org/10.1042/BJ20110366 21554250 10.1042/BJ20110366 PMC3863643

[acel12725-bib-0031] Peterson, C. M. , Johannsen, D. L. , & Ravussin, E. (2012). Skeletal muscle mitochondria and aging: A review. Journal of Aging Research, 2012, 194821. 22888430 10.1155/2012/194821 PMC3408651

[acel12725-bib-0032] Porter, C. , Hurren, N. M. , Cotter, M. V. , Bhattarai, N. , Reidy, P. T. , Dillon, E. L. , … Borsheim, E. (2015). Mitochondrial respiratory capacity and coupling control decline with age in human skeletal muscle. American Journal of Physiology. Endocrinology and Metabolism, 309, E224–E232. https://doi.org/10.1152/ajpendo.00125.2015 26037248 10.1152/ajpendo.00125.2015 PMC4525111

[acel12725-bib-0033] Reznick, R. M. , Zong, H. , Li, J. , Morino, K. , Moore, I. K. , Yu, H. J. , … Shulman, G. I. (2007). Aging‐associated reductions in AMP‐activated protein kinase activity and mitochondrial biogenesis. Cell Metabolism, 5, 151–156. https://doi.org/10.1016/j.cmet.2007.01.008 17276357 10.1016/j.cmet.2007.01.008 PMC1885964

[acel12725-bib-0034] Robinson, M. M. , Dasari, S. , Konopka, A. R. , Johnson, M. L. , Manjunatha, S. , Esponda, R. R. , … Nair, K. S. (2017). Enhanced protein translation underlies improved metabolic and physical adaptations to different exercise training modes in young and old humans. Cell Metabolism, 25, 581–592. https://doi.org/10.1016/j.cmet.2017.02.009 28273480 10.1016/j.cmet.2017.02.009 PMC5423095

[acel12725-bib-0035] Sandri, M. , Sandri, C. , Gilbert, A. , Skurk, C. , Calabria, E. , Picard, A. , Walsh, K. , … Goldberg, A. L. (2004). Foxo transcription factors induce the atrophy‐related ubiquitin ligase atrogin‐1 and cause skeletal muscle atrophy. Cell, 117, 399–412. https://doi.org/10.1016/S0092-8674(04)00400-3 15109499 10.1016/s0092-8674(04)00400-3 PMC3619734

[acel12725-bib-0036] Santanasto, A. J. , Coen, P. M. , Glynn, N. W. , Conley, K. E. , Jubrias, S. A. , Amati, F. , … Newman, A. B. (2016). The relationship between mitochondrial function and walking performance in older adults with a wide range of physical function. Experimental Gerontology, 81, 1–7. https://doi.org/10.1016/j.exger.2016.04.002 27084585 10.1016/j.exger.2016.04.002 PMC5483943

[acel12725-bib-0037] Shock, N. W. , Greulich, R. C. , Andres, R. A. , Arenberg, D. , Costa, P. T. , Lakatta, E. G. , … Tobin, J. D. (1984). Normal human aging: The Baltimore longitudinal study of aging (661 pp.). Washington, DC: US Government Printing Office; 1984.

[acel12725-bib-0038] Simonsick, E. M. , Montgomery, P. S. , Newman, A. B. , Bauer, D. C. , & Harris, T. (2001). Measuring fitness in healthy older adults: The Health ABC Long Distance Corridor Walk. Journal of the American Geriatrics Society, 49, 1544–1548. https://doi.org/10.1046/j.1532-5415.2001.4911247.x 11890597 10.1046/j.1532-5415.2001.4911247.x

[acel12725-bib-0039] Stone, J. L. , & Norris, A. H. (1966). Activities and attitudes of participants in the Baltimore longitudinal study. Journal of Gerontology, 21, 575–580. https://doi.org/10.1093/geronj/21.4.575 5918312 10.1093/geronj/21.4.575

[acel12725-bib-0040] Su, J. , Ekman, C. , Oskolkov, N. , Lahti, L. , Strom, K. , Brazma, A. , … Hansson, O. (2015). A novel atlas of gene expression in human skeletal muscle reveals molecular changes associated with aging. Skeletal Muscle, 5, 35 https://doi.org/10.1186/s13395-015-0059-1 26457177 10.1186/s13395-015-0059-1 PMC4600214

[acel12725-bib-0041] Tyrrell, D. J. , Bharadwaj, M. S. , Van Horn, C. G. , Kritchevsky, S. B. , Nicklas, B. J. , & Molina, A. J. A. (2015). Respirometric profiling of muscle mitochondria and blood cells are associated with differences in gait speed among community‐dwelling older adults. Journals of Gerontology. Series A, Biological Sciences and Medical Sciences, 70, 1394–1399. https://doi.org/10.1093/gerona/glu096 10.1093/gerona/glu096 PMC4731403 25030980

[acel12725-bib-0042] Zabielski, P. , Lanza, I. R. , Gopala, S. , Heppelmann, C. J. H. , Bergen, H. R. 3rd , Dasari, S. , & Nair, K. S. (2016). Altered skeletal muscle mitochondrial proteome as the basis of disruption of mitochondrial function in diabetic mice. Diabetes, 65, 561–573. https://doi.org/10.2337/db15-0823 26718503 10.2337/db15-0823 PMC4764144

[acel12725-bib-0043] Zane, A. C. , Reiter, D. A. , Shardell, M. , Cameron, D. , Simonsick, E. M. , Fishbein, K. W. , … Ferrucci, L. (2017). Muscle strength mediates the relationship between mitochondrial energetics and walking performance. Aging Cell, 16(3), 461–468. https://doi.org/10.1111/acel.12568 28181388 10.1111/acel.12568 PMC5418194

