# Supplementary material for: Skeletal muscle ex vivo mitochondrial respiration parallels decline in vivo oxidative capacity, cardiorespiratory fitness, and muscle strength: The Baltimore Longitudinal Study of Aging
Source: Aging Cell. 2018 Jan 21;17(2):e12725. doi: 10.1111/acel.12725 (PMC5847858; doi:10.1111/acel.12725)
Supplement: Supplementary file 1 [file ACEL-17-e12725-s001.pptx]

## Slide 1
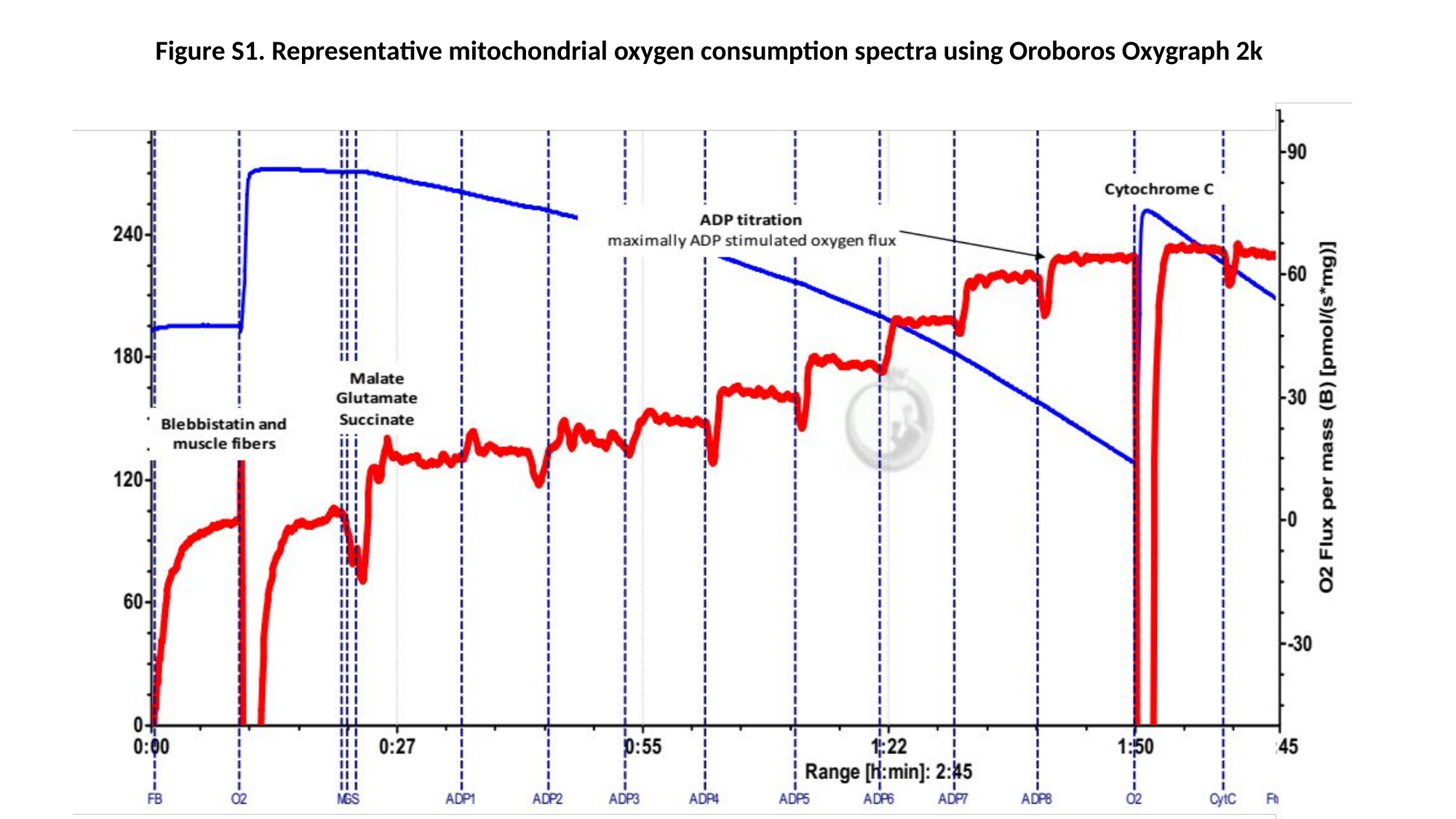

Figure S1. Representative mitochondrial oxygen consumption spectra using Oroboros Oxygraph 2k

## Slide 2
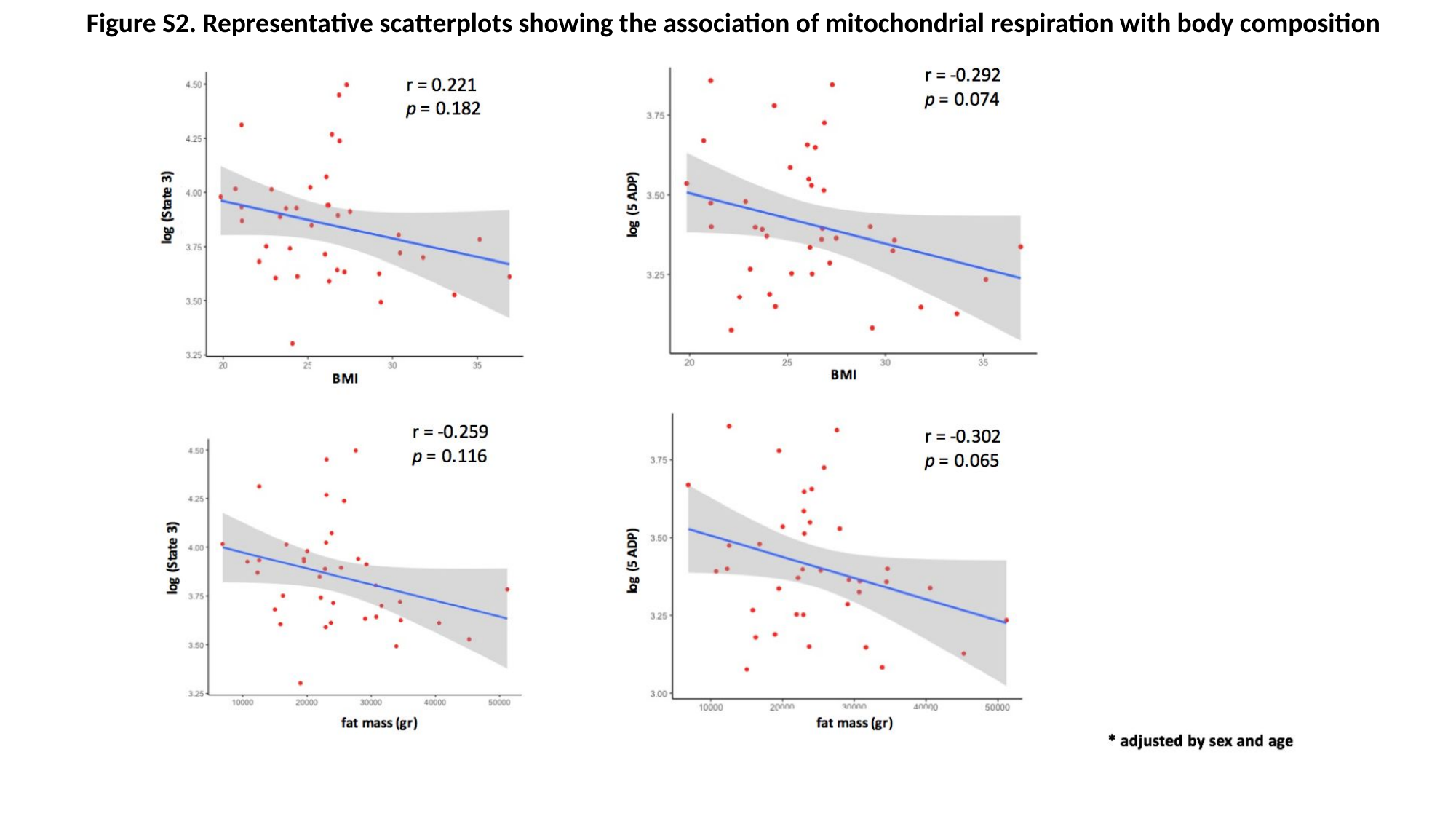

Figure S2. Representative scatterplots showing the association of mitochondrial respiration with body composition

## Slide 3
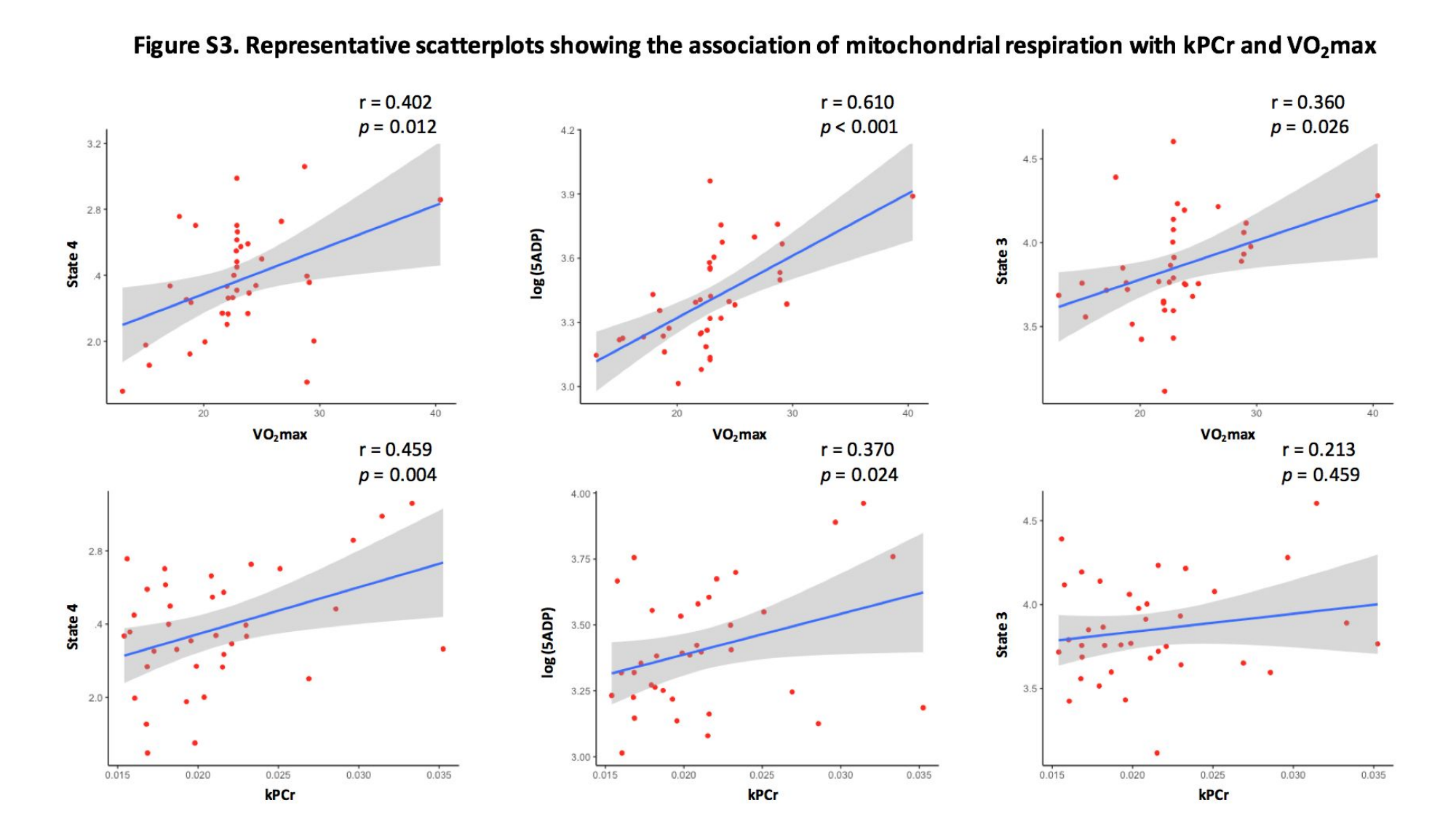

## Slide 4
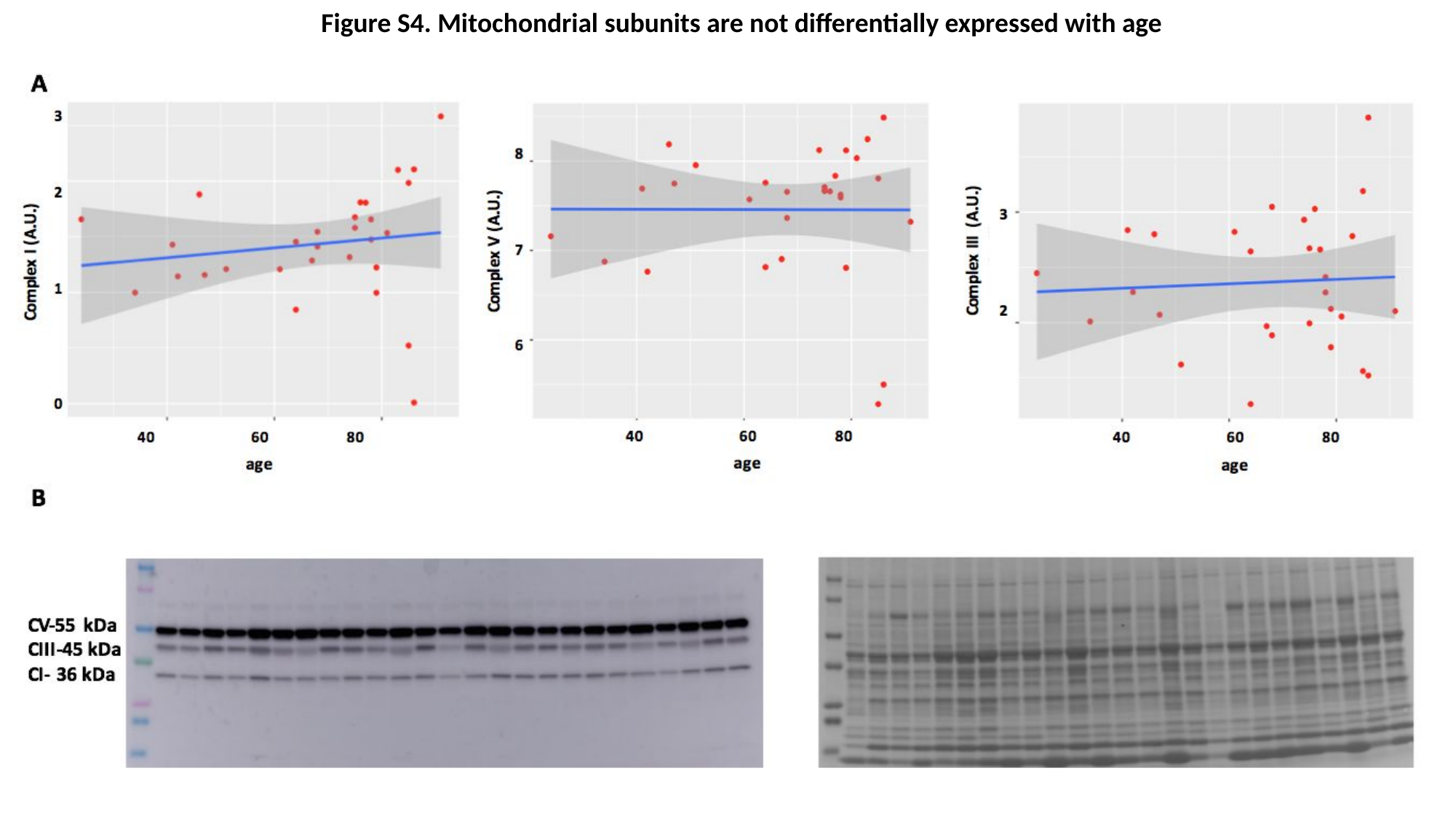

Figure S4. Mitochondrial subunits are not differentially expressed with age
