# Supplementary material for: Skeletal muscle ex vivo mitochondrial respiration parallels decline in vivo oxidative capacity, cardiorespiratory fitness, and muscle strength: The Baltimore Longitudinal Study of Aging
Source: Aging Cell. 2018 Jan 21;17(2):e12725. doi: 10.1111/acel.12725 (PMC5847858; doi:10.1111/acel.12725)
Supplement: Supplementary file 2 [file ACEL-17-e12725-s002.docx]

**Table S1**. Oxygen flux or mitochondrial oxygen consumption after the addition of different substrates

| **Titration** | **Oxygen flux**  **(pmol O_2_∙s^-1^∙mg^-1^ )** |
| --- | --- |
| LEAK state (malate, glutamate, succinate) -known as State 4 | 93.13 ± 30.74 |
| 1 ADP (10mM ADP**)_i_ [31.25µM]_m__31.25µM_f_** | 128.20 ± 35.07 |
| 2 ADP (10mM ADP)_i_ [31.25 µM] _m__62.5µM_f_ | 143.36 ± 40.57 |
| 3 ADP (10mM ADP)_i_ [62.5 µM] _m__125µM_f_ | 168.60 ± 45.48 |
| 4 ADP (10mM ADP)_i_ [125 µM] _m__250µM_f_ | 207.50 ± 55.73 |
| 5 ADP (50mM ADP)_i_ [250 µM] _m__500µM_f_ | 257.32 ± 63.48 |
| 6 ADP (500mM ADP)_i_ [500 µM] _m__1000µM_f_ | 316.54 ± 81.47 |
| 7 ADP (500mM ADP)_i_ [1000 µM] _m__2000µM_f_ | 376.27 ± 108.04 |
| 8 ADP (500mM ADP)_i_ State 3 [2000µM]_m__4000µM_f_ | 407.42 ± 127.45 |
| Area Under the Curve (AUC) (A.U.) | 1109.64 ±268.73 |

*Notes:* Values are mean ± standard deviation (SD); ADP= adenosine diphosphate; mM= millimolar; µM= micromolar; **i= stock solution; m= molarity added; f= final concentration of ADP in the chambers**

**Table S2.** **Multiple regression models showing the association between mitochondrial function and cardiorespiratory fitness, muscle function and in vivo oxidative capacity. All coefficients are standardized.**

| **Outcome** | **VO_2max_** | | **Grip Strength** | | **Left Leg muscle**  **Strength** | | **kPCr** | | **Time in 400 m** | |  |  |
| --- | --- | --- | --- | --- | --- | --- | --- | --- | --- | --- | --- | --- |
|  | **β (95% CI)** | **p-value** | **β (95% CI)** | **p-value** | **β (95% CI)** | **p-value** | **β (95% CI)** | **p-value** | **β (95% CI)** | **p-value** |  |  |
| **Model 1 #**  **(State 4)** | 0.252  (0.037,0.626) | 0.028* | 0.111  (-0.401, 1.145) | 0.334 | 0.116  (-1.828, 5.003) | 0.351 | 0.390  (0.000,0.0009) | 0.017* | -0.230  (-6.184, 0.684) | 0.113 |  |  |
| **Model 2 #**  **(5 ADP)** | 0.297  (0.028,0.350) | 0.023* | -0.025  (-0.471, 0.390) | 0.848 | 0.184  (-0.639, 3.068) | 0.192 | 0.264  (0.000, 0.0004) | 0.180 | -0.214  (-3.122, 0.670) | 0.197 |  |  |
| **Model 3 #**  **(State 4)** | 0.030  (0.073,0.092) | 0.812 | -0.063  (-0.254, 0.152) | 0.611 | 0.134  (-0.444, 1.328) | 0.318 | 0.057  (-0.0001, 0.0001) | 0.760 | 0.013  (-0.881,0.956) | 0.934 |  |  |

# all the models adjusted by age, sex and BMI * statistically significant

**Table S4, related to Figure 3C.** Top 10 downregulated genes present in the GO Term ‘GO0005746 Mitochondrial Respiratory Chain’ that were shared by the MA_Y and O_Y pairwise comparisons. Significance is defined as zratio > 1.5 in either direction, false discovery rate (fdr) < 0.3 and p < 0.05. N = 3 young, 4 middle-aged, and 12 old vastus literalis muscles.
