# Supplementary material for: Skeletal muscle ex vivo mitochondrial respiration parallels decline in vivo oxidative capacity, cardiorespiratory fitness, and muscle strength: The Baltimore Longitudinal Study of Aging
Source: Aging Cell. 2018 Jan 21;17(2):e12725. doi: 10.1111/acel.12725 (PMC5847858; doi:10.1111/acel.12725)
Supplement: Supplementary file 5 [file ACEL-17-e12725-s005.docx]

*Microarray accession link

<https://www.ncbi.nlm.nih.gov/geo/query/acc.cgi?token=ezkdmaqypxmhted&acc=GSE98613>
